# Headache service quality in Egypt: current status, identified gaps, and strategic directions

**DOI:** 10.3389/fneur.2026.1860601

**Published:** 2026-07-02

**Authors:** Amr Hassan, Mona Hussein, Rehab Magdy, Derya Uludüz, Aynur Özge, Semih Taşdelen, Sarkhan Amirguliyev, Tayyar Şaşmaz, Mohamad Osama Abdulghani, Ramez Reda Moustafa, Ahmed Essmat, Osama Yacoub, Mohamed Abdelghaffar, Nourhan Abdelmohsen Taha, Anas Elgenidi, Nahla Merghany, Sherien Mohamed Farag, May M. Fayez, Doaa Mahmoud Khalil, Rami Burstein

**Affiliations:** 1 Department of Neurology, Cairo University, Cairo, Egypt; 2 Department of Neurology, Beni-Suef University, Beni-Suef, Egypt; 3 Department of Neurology, Medical Faculty, Istanbul Atlas University, Istanbul, Türkiye; 4 Headache Clinic, Mersin, Türkiye; 5 NOROM Neuroscience and Excellence Center, Ankara, Türkiye; 6 Department of Neurology, İstanbul Faculty of Medicine, İstanbul University, Istanbul, Türkiye; 7 Azerbaijan State Institute of Advanced Training of Doctors Named After Aziz Aliyev, Baku, Azerbaijan; 8 School of Medicine, Mersin University, Mersin, Türkiye; 9 Department of Neurology, Ain Shams University, Cairo, Egypt; 10 Department of Neurology, Al Azhar University, Cairo, Egypt; 11 Department of Neurology, Fayoum University, Fayoum, Egypt; 12 Department of Neurology, El Mounira Hospital, Ministry of Health, Cairo, Egypt; 13 Department of Public Health and Community Medicine, Beni-Suef University, Beni-Suef, Egypt; 14 Department of Anaesthesia, Critical Care and Pain Medicine, Deaconess Medical Center, Boston, MA, United States; 15 Department of Anesthesia, Harvard Medical School, Boston, MA, United States

**Keywords:** Egypt, low- and middle-income countries (LMICs), medication access, provider training, structured headache services

## Abstract

**Background:**

Headache disorders are a leading cause of disability worldwide, yet service delivery in low- and middle-income countries (LMICs) like Egypt remains understudied. This study aimed to assess the national landscape of headache care in Egypt, focusing on provider training, treatment access, medication availability, and systemic disparities.

**Methods:**

This cross-sectional study is part of a larger multinational initiative conducted under the auspices of the International Headache Society (IHS). The study included 321 Egyptian neurologists. The data collection process was carried out through both electronic surveys and in-person interviews. The surveys included questions covering the following items: profile of headache patients, characteristics of healthcare services available for headache patients, headache medication choices and affordability, and perceived barriers to proper headache management.

**Results:**

Based on the neurologists’ perspective, the primary headaches accounted for 85% (65–90%) of all headache cases, whereas secondary headaches accounted for 10% (5–20%) of headache cases. About 91.9% of the neurologists reported that anxiety was the most common comorbidity with headache, followed by depression (81.9%). Approximately 80.1% (*n* = 257) of patients had to pay to access medicine, and the estimated percentage of patients who could afford medication was 60 (40–70). The average number of headache patients assessed by each specialist per week was estimated to be 25 (10–50) patients. About 89.7% (*n* = 288) of the participants reported receiving formal training on the diagnosis and treatment of migraine. Seeking medical advice from non-specialized physicians was reported by 82.9% of the included participants to be one of the commonest causes for the delayed diagnosis of headache. Most participants (76.6%) reported that financial constraints may be a contributing factor to non-compliance with medications in patients with headaches.

**Conclusion:**

This study identifies urgent gaps in the equity, affordability, and structure of headache care in Egypt. Policy action is needed to implement structured headache services, expand provider training, and improve access to essential and newly developed medications.

## 1 Introduction

Headache disorders, particularly migraine, are among the most prevalent and disabling neurological conditions worldwide, ranked second in global years lived with disability, according to the Global Burden of Disease study (1). Despite this burden, the delivery of effective and equitable headache care remains inconsistent, especially in low- and middle-income countries (LMICs), where health system limitations and medication access barriers, and uneven distribution of specialist care persist (2).

Egypt, the most populous country in the Middle East and North Africa, faces numerous challenges in diagnosing and treating headache patients. Despite the high prevalence of headaches among the Egyptian population (3–5), the subspecialty of headache, unlike more established neurological subspecialties, is still beginning to evolve. This is mostly due to the relatively small number of headache specialists. Specialized headache centres are also limited, mostly concentrated in urban areas, making access to headache specialists difficult for many patients, especially those in rural areas. This mismatch between the large number of patients and the availability of headache specialists exposes patients to inaccurate diagnosis and inappropriate treatment approaches (6).

Unlike LMICs, where clinical officers, nurses and pharmacists are authorized to diagnose headaches and initiate treatment, the Egyptian healthcare system strictly reserves the responsibility of diagnosis and treatment for licensed physicians. This rigid structure, while ensuring that only physicians diagnose and treat headache disorders, may also place an immense burden on the limited number of Neurologists (7).

In addition to structural issues, financial constraints play a significant role in impeding headache care in Egypt. A large portion of the Egyptian population lacks adequate health insurance coverage and consequently cannot afford newer migraine medications and if justified, basic brain imaging. In Egypt, most of the population is treated in public healthcare facilities that traditionally struggle with resource shortages, and while in the private sector, resources are far more available, they remain unaffordable for most patients. This financial barrier not only delays diagnosis and treatment but also discourages patients from seeking follow-up care (8).

Despite these challenges, efforts have been made to improve healthcare for headache patients in Egypt through training programs for primary care physicians and launching public awareness campaigns. However, without structural reforms, expanded insurance coverage, and better integration of trained healthcare professionals into the system, achieving comprehensive headache care in Egypt will remain challenging (9).

This study aims to explore the profile of headache patients in Egypt, the pros and cons of the healthcare system provided to them from the neurologists’ perspective, and the key challenges faced by physicians that impede the delivery of optimal medical care to these patients.

## 2 Methods

### 2.1 Study design and setting

This study is part of a larger multinational initiative conducted under the auspices of the International Headache Society (IHS), aiming to evaluate access to proper headache care in LMICs. Specifically, this research focuses on Egypt and assesses the current landscape of headache care, service availability, and barriers to optimal management. Data were collected using a modified version of the International Headache Society’s Headache-Attributed Restriction, Disability, Social Handicap, and Impaired Participation (HARDSHIP) questionnaire, which served as a conceptual framework for developing questions related to headache service delivery, access to care, medication affordability, and healthcare barriers. The original questionnaire was adapted to reflect the characteristics of the Egyptian healthcare system and local clinical practice. Modifications included the addition of items related to provider training, access pathways, availability of interventional headache management, and affordability of acute and preventive headache medications.

The survey instrument was adapted to ensure contextual appropriateness for Egypt, taking into account linguistic, cultural, and healthcare infrastructure differences. The adapted survey was reviewed by Egyptian neurologists experienced in headache medicine and public health to ensure linguistic clarity, contextual relevance, and cultural appropriateness. Minor wording modifications were introduced where necessary to improve comprehensibility in the local clinical setting. Prior to nationwide dissemination, pilot testing was conducted on a small group of neurologists, and ambiguous or unclear items were revised accordingly. Because the survey consisted mainly of descriptive and service-oriented items rather than psychometric composite scales, formal internal consistency analyses, such as Cronbach’s alpha, were not considered methodologically appropriate for most sections of the questionnaire.

Another questionnaire was sent electronically to participants to collect additional data on healthcare services available to headache patients in Egypt and the major barriers faced by Egyptian neurologists in headache management, taking into account the unique aspects of the national healthcare system and headache services.

### 2.2 Participant recruitment and data collection

The study population included 321 Egyptian Neurologists. Eligible participants were licensed Egyptian neurologists actively involved in the diagnosis and management of headache disorders in clinical practice. Neurologists from university hospitals, Ministry of Health hospitals, insurance hospitals, general hospitals, and private clinics were invited to participate through electronic distribution of the survey and direct in-person recruitment during professional meetings and clinical activities. Non-Egyptian neurologists or neurologists who were not actively involved in the diagnosis and management of headache disorders were excluded from the final analysis.

Although the study population exclusively consisted of neurologists practicing headache management in Egypt, several international co-authors participated in the study design, methodological supervision, survey framework development, and data interpretation as part of the broader IHS collaborative initiative.

### 2.3 Surveys components and variables

The two surveys included questions covering the following items:

- *Profile of headache patients in Egypt* regarding age, gender, education, economic level, affordability of medications, preferred hospitals/clinics for headache patients to seek medical advice, specialties other than Neurology who treat headache patients, types, and common comorbidities associated with headache. The age-group categorization of the patients was designed to reflect clinically relevant developmental stages commonly considered in headache practice, with narrower categories for pediatric, adolescent, and young adult populations, given their distinct clinical characteristics, healthcare access patterns, and management approaches. Participants older than 25 years were grouped together to provide a broader overview of adult headache service utilization patterns.
- *Characteristics of healthcare service available for headache patients in Egypt*: Average number of headache patients assessed by each specialist per week, formal training on diagnosis and treatment of migraine headache, waiting lists for headache patients to consult a headache specialist/Neurologist, availability of brain imaging, and availability of interventional pain management for headache patients.
- *Headache medication choices and affordability*: The most prescribed medications for headache patients, and the availability of medications to those who can afford and those who cannot.
- *Perceived barriers to headache management*: cause of delayed diagnosis of headache, percentage of patients compliant with medications, and reasons for non-compliance.

### 2.4 Ethical considerations

The study complied with the ethical guidelines established by the IHS and the MENAA Headache Group. It was supported by a general ethical approval in Turkey as the coordinating country (24.12.2024/218). Additionally, the study was ethically approved by the Faculty of Medicine, Beni-Suef University Research Ethics Committee (FM-BSU-REC), with approval number: FMBSUREC/01072025/Khalil2. All participants provided informed consent before data collection, and anonymity and confidentiality were strictly maintained.

### 2.5 Sample size calculation

The minimum required sample size was calculated using EpiInfo StatCalc at a 95% confidence level, 5% margin of error, one design effect, and an expected frequency of 80% (neurologists managing headache cases) from a total population of 4,000 neurologists as reported by Kissani et al. (6). The result indicated a minimum sample size of 232 neurologists. To enhance representativeness and minimize selection bias, we increased the target sample size to 321 neurologists.

### 2.6 Data management and statistical analysis

Survey responses were compiled and analysed using SPSS Statistics 26.0. The Kolmogorov–Smirnov test was used to assess the normality of data distribution. Highly skewed variables were reported as median and interquartile range (IQR). No inferential statistical tests were performed, as the study was designed to provide a descriptive overview of clinicians’ perspectives on headache care in Egypt rather than to conduct comparative group analyses.

Missing data were handled by listwise deletion, with incomplete responses excluded from the relevant analyses. Sensitivity checks confirmed that exclusion did not materially affect the descriptive estimates.

## 3 Results

### 3.1 Profile of headache patients in Egypt

**Figure 1:**
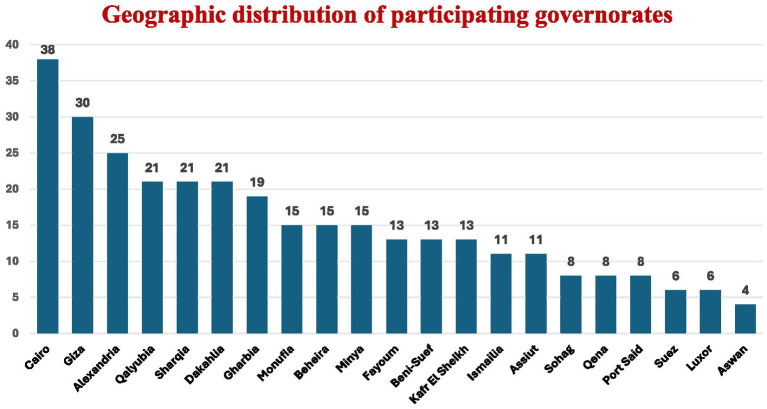
Geographic distribution of participating governorates.

A total of 321 Egyptian Neurologists participated in the survey (157 males and 164 females), with a median age of 33 (30–39) years. The included participants were recruited from tertiary and secondary hospitals located in major urban centers across both the public and private healthcare sectors. The geographic distribution of the participating neurologists across Egyptian governorates was demonstrated in Figure 1.

The estimated percentages of age, sex, educational, and economic levels of headache patients, as reported by the included neurologists, are demonstrated in Table 1. The participants reported that about 80.1% of patients have to pay to access medicine, and the estimated percentage of patients who can afford medication was 60 (40–70). According to 37.7% of the participants, the private neurology clinics were the most preferred setting for headache patients to seek medical advice, followed by university hospitals (26.5%), general hospitals (19.3%), insurance hospitals (8.4%), and primary care centres (8.1%).

**Table 1:** Profile of headache patients in Egypt.

Variable	Participants (*n* = 321)
Estimated percentage of patients who are [median (IQR)]^1^	
0–5 years old (%)	5 (1–10)
6–11 years old (%)	10 (5–10)
12–17 years old (%)	10 (10–20)
18–25 years old (%)	20 (15–30)
25 and older (%)	50 (37.5–60)
Estimated percentage of patients who are [median (IQR)]^a^	
Women	60 (60–70)
Men	40 (30–40)
Estimated education level of those served by headache clinic [median (IQR)]^a^	
Below high school (%)	30 (20–50)
High school (%)	25 (20–30)
College (%)	25 (12.5–40)
Post-graduate (%)	10 (9–20)
Estimated economic level of those served by headache clinic [median (IQR)]^a^	
Income is below my expenses (Below poverty line) (e.g., earning $1/day/household)	30 [20–50]
Income covers my expenses (Low income %) (e.g., earning $5/day/household)	30 [20–50]
Income is slightly more than my expenses (Middle income %) (e.g., earning $10/day/household)	20 (15–30)
Income is above my economic needs (High income %) (e.g., earning $20/day/household)	10 (5–20)
Patients who have to pay to access medicine [*n* (%)]	
Yes	257 (80.1%)
No	64 (19.9%)
Estimated percentage of patients who can afford medication [median (IQR)]^a^	
Those who can pay	60 (40–70)
Those who cannot pay	40 (25–60)
The preferred hospitals/clinics for headache patients to seek medical advice [*n* (%)]	
Primary care centres	26 (8.1%)
Insurance hospitals	27 (8.4%)
University hospitals	85 (26.5%)
General hospitals	62 (19.3%)
Private clinics	121 (37.7%)
Other medical specialties other than neurology that headache patients may consult [*n* (%)]	
Ophthalmology	107 (33.3%)
ENT	86 (26.8%)
Internal medicine	117 (36.4%)
Dentistry	11 (3.4%)
Estimated percentage of the types of headache [median (IQR)]	
Primary headache	85% (65–90%)
Secondary headache	10% (5–20%)
Common comorbidities associated with headache [*n* (%)]^b^	
Anxiety	295 (91.9%)
Depression	263 (81.9%)
Fibromyalgia	189 (58.9%)
IBS	191 (59.5%)
HTN	198 (61.7%)
Hypotension	134 (41.7%)
Anemia	200 (62.3%)

ENT, Ear, Nose, and Throat; HTN, hypertension; IBS, irritable bowel syndrome.

a Percentages represent neurologists’ estimated proportions of patient characteristics encountered in routine clinical practice rather than mutually exclusive categorical distributions.

b Participants were allowed to select more than one response option.

The participants reported that the most frequently consulted specialty (other than neurology) for headache patients was internal medicine (36.4%), followed by ophthalmology (33.3%), Ear, Nose, and Throat (ENT) (26.8%), and dentistry (3.4%).

Based on the neurologists’ perspective, the primary headaches accounted for 85% (65–90%) of all headache cases, whereas secondary headaches accounted for 10% (5–20%) of headache cases. About 91.9% of the neurologists reported that anxiety was the most common comorbidity with headache, followed by depression (81.9%), anemia (62.3%), hypertension (61.7%), irritable bowel syndrome (IBS) (59.5%), fibromyalgia (58.9%), and hypotension (41.7%).

### 3.2 Characteristics of healthcare service available for headache patients in Egypt

The average number of headache patients assessed by each specialist per week was estimated to be 25 (10–50) patients. Approximately 89.7% (*n* = 288) of the participants (notably predominantly neurologists) reported having received formal training in the diagnosis and treatment of migraine headaches. Only 22.1% of the included participants reported that their institutions have waiting lists for patients with headaches to consult a headache specialist, and 61.7% reported that there are waiting lists for brain imaging for these patients. Interventional pain management (botulinum toxin injection, nerve block, and neuromodulation techniques) was reported by 55.5% of the participants to be available in their institutes for headache patients (Table 2).

**Table 2:** Characteristics of healthcare service available for headache patients in Egypt.

Variable		Participants (*n* = 321)
Average number of headache patients assessed by each specialist per week [median (IQR)]		25 (10–50)
Formal training on diagnosis and treatment of migraine headache [*n* (%)]	Yes	288 (89.7%)
	No	33 (10.3%)
Waiting lists for headache patients to consult a headache specialist [*n* (%)]	Yes	71 (22.1%)
	No	250 (77.9%)
Waiting lists for brain imaging for headache patients [*n* (%)]	Yes	198 (61.7%)
	No	123 (38.3%)
Availability of interventional pain management for headache patients [*n* (%)]	Yes	178 (55.5%)
	No	143 (44.5%)

### 3.3 Medication prescription and affordability

As shown in Table 3, the most frequently prescribed medications were paracetamol (86.6%), topiramate (76.6%), triptans (76.0%), and amitriptyline (74.8%), indicating a tendency toward evidence-based pharmacological management for both acute and chronic migraine. However, a significant gap exists in availability and affordability, particularly for newer or more expensive medications. While 91.0% of the participants reported paracetamol as accessible to patients who cannot afford treatment, this number drops sharply to 41.1% for triptans and as low as 7.8% for CGRP-targeting drugs.

**Table 3:** Headache medication choices and affordability.

Medication	Most prescribed		Available to those who can pay		Available to those who cannot pay	
	*N* (%)		*N* (%)		*N* (%)	
Paracetamol	278	86.6	302	94.1	292	91.0
NSAIDS	221	68.8	278	86.6	271	84.4
Triptans	244	76.0	259	80.7	132	41.1
Ergots	75	23.4	165	51.4	108	33.6
CGRP Monoclonal antibodies	67	20.9	126	39.3	25	7.8
Ditans	9	2.8	51	15.9	18	5.6
Gepants	46	14.3	88	27.4	29	9.0
Onabotulinumtoxin A	59	18.4	125	38.9	28	8.7
Valproate	134	41.7	222	69.2	150	46.7
Topiramate	246	76.6	267	83.2	164	51.1
Propranolol	236	73.5	263	81.9	197	61.4
Metoprolol	38	11.8	149	46.4	88	27.4
Amitriptyline	240	74.8	254	79.1	183	57.0
Candesartan	65	20.2	150	46.7	76	23.7
Lisinopril	18	5.6	141	43.9	72	22.4
Cyproheptadine	57	17.8	135	42.1	75	23.4
Venlafaxine	86	26.8	188	58.6	62	19.3
Gabapentin	121	37.7	223	69.5	126	39.3
Duloxetine	133	41.4	214	66.7	90	28.0

CGRP, Calcitonin Gene-Related Peptide; NSAIDS, non-steroidal anti-inflammatory drugs.

Participants were allowed to select more than one response option.

Preventive medications also show discrepancies: Valproate, topiramate, and propranolol were widely accessible and prescribed, whereas other agents, such as candesartan (available to only 23.7% of low-income patients) and duloxetine (28.0%), were less accessible.

### 3.4 Perceived barriers to headache management in Egypt

As shown in Table 4, barriers to proper headache care are attributed to patients seeking medical advice from non-neurologists, lack of awareness, common use of simple analgesics, wrong medical information from online sources or social media, and financial difficulties. Leading reasons for lack of compliance with prescribed medications included financial difficulties, unwanted side effects, and a poor response to prescribed medications.

**Table 4:** Perceived barriers to headache management in Egypt.

Variable		Participants (*n* = 321)
Cause of delayed diagnosis of headache [*N* (%)]	Financial constraints	218 (67.9%)
	Lack of awareness	262 (81.6%)
	Seeking medical advice from non-neurologists	266 (82.9%)
	Response of most headache types to simple analgesics	232 (72.3%)
	Wrong medical information from online sources or social media	196 (61.1%)
Percentage of patients compliant with medications [median (IQR)]		50% (35–60%)
Reasons for lack of compliance of some headache patients with medications [*N* (%)]	Financial constraints	246 (76.6%)
	Side effects from medications	215 (67.0%)
	Poor response to the prescribed medications	198 (61.7%)

Participants were allowed to select more than one response option.

## 4 Discussion

This study aimed to conduct the first nationwide evaluation of headache care delivery in Egypt, assessing provider characteristics, treatment practices, and access to medication across various healthcare settings. As part of the IHS initiative, this research provides a foundational dataset for understanding the real-world landscape of headache management in a low- and middle-income country (LMIC) context.

Physicians’ insights into patient demographics were consistent with prior epidemiological reports conducted in Egypt (3, 5, 10) in terms of the predominance of primary headache disorders (85%) and the female gender (approximately 62%). Recognizing the clinical and economic profile of patients is mandatory, as it enables the development of appropriate treatment strategies.

In the present study, the surveyed neurologists believed that the prevalence of depression and anxiety among headache patients in Egypt is two to three times higher than the rates reported in previous meta-analyses, in which anxiety is roughly estimated at 25–35% and depression at 20–30% (11–13). Such a discrepancy raises the possibility of information bias by the surveyed neurologists, as their estimates were based on subjective clinical impressions rather than systematic psychiatric assessment. In addition, the participating neurologists were mainly working in specialized headache centers, where more severe, treatment-resistant, and highly disabled patients are more likely to be encountered, potentially leading to an overestimation of psychiatric comorbidities compared with population-based studies.

The current survey revealed that the most common medical specialties other than neurology that a headache patient might consult are ophthalmology, ENT, and internal medicine. Genc, Uluduz (14) found that about 15.07% of headache patients were referred to neurology clinics by other specialties. Identifying these specialties that a headache patient may consult is crucial in promoting multidisciplinary collaboration, developing effective referral algorithms and eventually improving headache care service.

On the positive side, the study showed that nearly all (90%) Egyptian neurologists receive formal training in headache medicine. On the negative side, it became apparent that financial constraints prevent access to essential acute and chronic migraine medication. These findings reflect the concern raised by Hill, Reynolds (15) regarding the increasing out-of-pocket costs associated with neurological disorders. Medication affordability and a lack of universal insurance were consistently reported as a major barrier to headache healthcare in studies focusing on the low- and middle-income countries (8, 16). Regrettably, the financial issues were cited as the most common cause of medication non-adherence. Such financial constraints were also approved by a previous survey that relied on the perspectives of 8,346 migraine patients (17).

Notably, the frequent use of paracetamol and topiramate, as declared by the surveyed neurologists, is not adequately contextualized within current headache management guidelines, which prioritize triptans and NSAIDs for acute attacks. Yet, this over-reliance primarily stems from medication affordability and accessibility, rather than clinical preference. Unfortunately, the scarcity of available preventative headache medications and the ease of taking over-the-counter painkillers may contribute to high rates of medication-overuse headaches in Egypt (18), as is the case elsewhere in low- and middle-income countries (19). Of note, in an effort to reduce the financial barrier impact, the Egyptian government has recently taken steps toward establishing a new health insurance system (Egypt Vision 2030) to make healthcare services available to all residents, regardless of their income level (20).

On the other hand, the era of interventional headache management has expanded in Egypt, yielding fruitful results, ranging from the use of botulinum toxin type A (21, 22) to various nerve block modalities (23–26) and neuromodulation techniques (27, 28). However, only 55.5% of respondents approved the availability of interventional pain management for headache patients in their centres, suggesting the need for concerted efforts to make it more widely available and accessible to a broader range of patients.

The surveyed neurologist cited a lack of awareness among patients as a potential barrier to the proper diagnosis of headaches in Egypt. Such a finding may explain the high prevalence of patients self-treating with over-the-counter painkiller medications (29). Intriguingly, the study suggests that inaccurate medical information from online sources or social media contributes to the lack of awareness. Addressing this issue will require creating reliable medical platforms and organizing headache awareness campaigns to educate the public about headaches, dispel myths, enhance diagnosis, and promote early management.

Finally, the characteristics of headache healthcare in Egypt should be highlighted. First, the urban–rural disparity, as specialized neurology and headache services are concentrated mainly in urban areas, while in rural areas, services are largely provided by primary care physicians (PCPs) with limited access to neurologists. Activating telemedicine services and providing PCPs in rural areas with diagnostic questionnaires and treatment algorithms would reduce patient transportation rates and ensure healthcare equality access between urban and rural areas. Second, a dual-practice model in the Egyptian healthcare system, where neurologists are allowed to work simultaneously in public and the private healthcare sectors. Third, pharmacists and nurses are not legally authorized to diagnose or prescribe treatment. Therefore, the burden of diagnosis and prescribing treatment falls on the specialist physicians. Nevertheless, most physicians surveyed (77.9%) believed that there are no waiting lists for patients with headaches to consult a specialist in headaches. Although this system provides high-quality healthcare services to patients with headaches, it undoubtedly imposes a significant work burden on specialist physicians. An Egyptian study in 2020 (30) found that 86.8% of physicians worked more than 48 h per week, negatively impacting their psychological well-being and quality of life (31).

Ultimately, although this study offers valuable insights into the headache care landscape in Egypt, some limitations must be acknowledged. First, the study was limited to surveying neurologists and did not address the opinions of doctors in other medical specialties who also deal with headache cases. Second, it captures provider perceptions and practice patterns at a single point in time. Third, it was not conducted among patients, and as such, the providers’ perception could not be verified. Fourth, participating neurologists were recruited predominantly from tertiary and secondary hospitals located in major urban centers. This reflects the current structure of neurological healthcare delivery in Egypt, where neurologists and specialized headache services are mainly concentrated in urban areas, while rural regions are largely served by primary care physicians and general practitioners with limited access to neurology specialists. Consequently, rural healthcare settings may have been underrepresented in the present study, potentially limiting the generalizability of the findings to the entire Egyptian population.

A further limitation is the lack of comparisons between healthcare providers working in public and private settings. Such comparisons were not feasible in the current study because, within the Egyptian healthcare system, many neurologists practice simultaneously in both public hospitals and private clinics. Consequently, the boundaries between these practice settings are not clearly distinct, limiting the ability to perform clear-cut comparisons between the public and private healthcare sectors in this study.

## 5 Conclusion

This study presents the first comprehensive national assessment of neurologists’ views about access to headache care in Egypt. Despite the high rate of training among neurologists, widespread lack of public awareness and unaffordability of medicines remain major barriers to access to evidence-based treatments. The mismatch between drug prescribing patterns and availability underscores the need for greater coordination in policy actions.

## Data Availability

The raw data supporting the conclusions of this article will be made available by the authors, without undue reservation.
